# Identifying subtypes of trichotillomania (hair pulling disorder) and excoriation (skin picking) disorder using mixture modeling in a multicenter sample

**DOI:** 10.1016/j.jpsychires.2020.11.001

**Published:** 2021-05

**Authors:** Jon E. Grant, Tara S. Peris, Emily J. Ricketts, Christine Lochner, Dan J. Stein, Jan Stochl, Samuel R. Chamberlain, Jeremiah M. Scharf, Darin D. Dougherty, Douglas W. Woods, John Piacentini, Nancy J. Keuthen

**Affiliations:** a Department of Psychiatry & Behavioral Neuroscience University of Chicago, Chicago, IL, USA; b Department of Psychiatry and Biobehavioral Sciences, University of California, Los Angeles, CA, USA; c Department of Psychiatry, University of Stellenbosch, South Africa; d SA MRC Unit on Risk & Resilience in Mental Disorders, Dept of Psychiatry & Neuroscience Institute, University of Cape Town, South Africa; e Department of Psychiatry, University of Cambridge, Cambridge, UK; f Department of Kinanthropology, Charles University, Prague, Czech Republic; g Department of Psychiatry, Massachusetts General Hospital and Harvard Medical School, Boston, USA; h Department of Psychology, Marquette University, Milwaukee, WI, USA

**Keywords:** Trichotillomania, Skin picking disorder, Subtypes, Classification, Treatment, Mixture modeling

## Abstract

Body-focused repetitive behavior disorders (BFRBs) include Trichotillomania (TTM; Hair pulling disorder) and Excoriation (Skin Picking) Disorder (SPD). These conditions are prevalent, highly heterogeneous, under-researched, and under-treated. In order for progress to be made in optimally classifying and treating these conditions, it is necessary to identify meaningful subtypes. 279 adults (100 with TTM, 81 with SPD, 40 with both TTM and SPD, and 58 controls) were recruited for an international, multi-center between-group comparison using mixture modeling, with stringent correction for multiple comparisons. The main outcome measure was to examine distinct subtypes (aka latent classes) across all study participants using item-level data from gold-standard instruments assessing detailed clinical measures. Mixture models identified 3 subtypes of TTM (entropy 0.98) and 2 subtypes of SPD (entropy 0.99) independent of the control group. Significant differences between these classes were identified on measures of disability, automatic and focused symptoms, perfectionism, trait impulsiveness, and inattention and hyperactivity. These data indicate the existence of three separate subtypes of TTM, and two separate subtypes of SPD, which are distinct from controls. The identified clinical differences between these latent classes may be useful to tailor future treatments by focusing on particular traits. Future work should examine whether these latent subtypes relate to treatment outcomes, or particular psychobiological findings using neuroimaging techniques.

## 1 Introduction

Trichotillomania (TTM) and Excoriation (Skin Picking) Disorder (SPD), are characterized by repeated pulling out of one's hair resulting in hair loss and picking at one's skin resulting in tissue damage, respectively, and have been conceptualized as body focused repetitive behavior disorders (BFRBs). BFRBs often result in significant impairment in physical, social, and psychological domains, and they may exact an enormous personal and societal cost (Tucker et al., 2011; Walther et al., 2014). Both psychosocial and psychopharmacological treatments have demonstrated some degree of efficacy; however, many individuals fail to respond or exhibit only partial response to these interventions (Lee et al., 2019; Sani et al., 2019). Relapse is common over the long-term and remission is unusual (Franklin et al., 2011).

One issue that has hampered treatment development to date is that the understanding of the phenomenology and pathophysiology of BFRBs remains limited by the relatively small samples enrolled in most clinical studies. Existing literature suggests environmental and internal factors, including boredom, activity restriction, emotional reactivity, stress response, abnormalities in perceptual sensitivity, dissociation, and trauma history may all contribute to symptom provocation and exacerbation of BFRBs (for a review (Snorrason et al., 2012)). Both disorders are familial, and twin studies have demonstrated significant shared genetic risk between TTM and SPD, as well as shared heritability with obsessive-compulsive spectrum disorders more broadly (Monzani et al., 2014).

In terms of neurocognitive processing and brain networks in BFRBs, the literature reveals mixed findings from neurocognitive examinations designed to assess motor inhibition, spatial working memory, divided attention, visuo-spatial learning, and cognitive flexibility (Chamberlain et al., 2009; Snorrason et al., 2011; Francazio and Flessner, 2015).

Furthermore, BFRB research comparing patients to matched controls has identified abnormalities in multiple brain regions underpinning many of the above neurocognitive domains. In small samples of participants (Ns ranging from 10 to 76), there has been evidence for abnormalities in multiple brain regions, including those regions involved in habit learning (e.g., striatum), emotion regulation (e.g., amygdalo–hippocampal complex), memory processing (e.g., temporal lobe), self-monitoring and awareness (e.g., precuneus), reward processing (e.g., ventral striatum, frontal hemisphere, bilateral cuneus), visual processing of disgust (e.g., insula and putamen), and the ability to generate and suppress motor responses (e.g., several cortical areas, including the right frontal gyrus) (Chamberlain et al., 2009; Flessner et al., 2012; Chamberlain et al., 2018; Isobe et al., 2018).

These varied findings from phenomenological, cognitive, and neurobiological studies, as well as inconsistent results from psychosocial and pharmacological interventions (Franklin et al., 2011; Rothbart et al., 2013), suggest heterogeneity within these disorders. In fact, for years, clinical studies have attempted to parse the possible etiological and phenotypic heterogeneity in TTM and SPD based on a variety of parameters such as sex, pulling/picking style, pulling/picking triggers, age of onset, co-occurring mental health disorders, family history, affect regulation and emotional cues (Flessner et al., 2013; Pozza et al., 2016; Lochner et al., 2019; Ricketts et al., 2019). These findings, however, have been undermined by relatively small sample sizes and inconsistent findings, and integrated models of disease pathology have yet to emerge. The result is that there remains a substantive need for further work aimed at understanding the clinical, biological, and neurocognitive underpinnings of BFRBs.

A deeper understanding of phenotypic heterogeneity should improve efforts to clarify BFRB pathophysiology by identifying more homogeneous BFRB-related latent phenotypes. Mixture modeling (MM) is a type of statistical methodology combining latent profile analysis and latent class analysis that has been widely used to identify candidate subtypes of a variety of mental disorders, including eating disorders, depression, and post-traumatic stress disorder (Wade et al., 2006; Hansen et al., 2017). Essentially, MM identifies separable groups of individuals differentiated by values on an unobserved latent variable, constructed from multiple measured variables. The use of MM statistical approaches to identify subgroups of people with BFRBs based on patterns of symptom expression can provide evidence of novel phenotypic subtypes that may reflect the underlying neurocircuitry of these behaviors. This approach, in turn, should allow for improved prevention and treatment strategies tailored to the needs of individual profiles (Collins and Varmus, 2015). Thus, the objective of this study was to identify and characterize distinct latent subtypes of TTM and SPD among a large well characterized sample of diagnosed adults.

## 2 Materials and methods

Participants included 279 adults recruited from the community and identified as having either a BFRB (meeting DSM-5 criteria for TTM, SPD, or both as their primary psychiatric problem; if a person had DSM-5 TTM and some skin picking that did not meet full diagnostic criteria for SPD, they would be classified as having only TTM) or being a healthy control. Four sites were involved in recruitment: University of Chicago, University of California, Los Angeles, Massachusetts General Hospital/Harvard Medical School, and Stellenbosch University. Recruitment started in October 2017 and ended in March 2019.

Inclusion criteria for the clinical sample were: a) DSM-5 diagnosis of TTM and/or SPD; b) aged 18–65 years; c) fluency in English; and d) capable of providing informed consent. Inclusion criteria for the healthy controls were the same except they could have no current or lifetime history of any DSM-5 psychiatric disorder.

Exclusion criteria for the clinical sample and healthy controls were: (a) current or lifetime diagnosis of any serious medical or psychiatric illness that would preclude successful study participation (e.g., psychotic disorder, intellectual disability); (b) neurological conditions that would preclude completion of neurocognitive tasks; (c) use of psychotropic medications unless the dose had been stable for at least the past 3 months; (d) body metal other than dental fillings (assessed using a neuroimaging screening form) (this was because all participants were only enrolled at the US sites if they were also able to undergo neuroimaging); (e) positive pregnancy test for females of childbearing age; and (f) medical condition or other factor (e.g., vision or hearing problems) that would interfere in the subject's ability to participate in the study.

### 2.1 Procedures

Potential participants were screened by the study site coordinator, who then scheduled an interview date. On the day of the assessment, participants met with study staff to complete informed consent. They were given an opportunity to ask questions and were reminded that study participation was voluntary. The primary investigator and/or trained study personnel discussed potential risks of the study prior to obtaining informed consent. After receiving a complete description of the study, participants provided written informed consent. Participants received a cash incentive for participation to reimburse them for their time and transport costs.

The authors assert that all procedures contributing to this work comply with the ethical standards of the relevant national and institutional committees on human experimentation and with the Helsinki Declaration of 1975, as revised in 2008. All procedures involving human subjects were approved by the Institutional Review Boards at each of the participating universities. Data sharing agreements were arranged across all sites.

### 2.2 Assessments

All participants completed a comprehensive diagnostic interview (Mini International Neuropsychiatric Interview 7.0 (MINI 7.0) (Sheehan et al., 1998); BFRB diagnostic modules and symptom severity scales, which were completed by trained diagnosticians with a bachelor's degree or higher trained to reliability and supervised by a doctoral-level clinician; neurocognitive tasks from the Cambridge Neurocognitive Test Automated Battery (CANTAB; http://www.cantab.com), which were counter-balanced; and self-report questionnaires regarding BFRB symptoms, general psychopathology, quality of life, and family environment completed at home via a web link through Research electronic data capture (REDCap)(Harris et al., 2009). A detailed list of the assessments is provided in the Supplement. The total assessment time was approximately 4–5 h. Study participation could be divided into two visits scheduled across two consecutive days (no more than 14 days between visits), with breaks permitted if needed.

### 2.3 Quality assurance

Drawing on approaches used in other research where the integrity of diagnostic conclusions has been paramount and where there have been multiple domains of assessment, we tracked diagnostic assessment and clinical interview procedures; adherence to imaging and neurocognitive protocols; and adverse event prevention and response. Protocol fidelity monitoring was addressed by several mechanisms including written guidelines, monitoring forms, on-site supervision, and cross-site calls. Each site was led by an investigator with extensive clinical and research expertise in BFRBs. Cross-site panels monitored caseness. Ongoing monitoring took place via team meetings at each site weekly and cross-site teleconferences conducted regularly. Among other things, the cross-site calls were used to review interviews, and in the instance of diagnostic disagreement, the sources of these differences were discussed and a consensus diagnosis was reached.

### 2.4 Data analysis

Mixture modeling (MM) was used to identify a number of homogeneous, distinct subtypes (aka classes) across all study participants. To identify TTM subtypes, we used item-level data of the 13-item Milwaukee Inventory for Subtypes of Trichotillomania (MIST-A-R)(Keuthen et al., 2015), which assesses intentionality and emotionality, combined with item-level data from the Massachusetts General Hospital Hair Pulling Scale (MGH-HPS) (Keuthen et al., 2005), a 7-item severity scale assessing urges and resistance. To identify SPD subtypes, we used item-level data of the 12-item MIDAS (Milwaukee Inventory for the Dimensions of Skin Picking) (Walther et al., 2009), which assesses automatic and focused picking styles, combined with item-level data from the 8-item Skin Picking Scale-Revised (SPS-R) (Snorrason et al., 2012), a scale of severity based on urge and resistance. Each measure used has excellent psychometric properties. After several online meetings consensus was reached amongst international experts of BFRBs that these MM input variables were most reflective of the core validated symptoms of each disorder. We also ran sensitivity analysis where we estimated MM models (without predictors) for only clinical cases (see Supplement).

Maximum likelihood estimation with 100 random starts was used to minimize the risk of finding local maxima. We tested models with up to six classes. Selection of the optimal model was based on Bayesian Information Criteria (BIC) (Akaike, 1974; Schwarz, 1978) (we report both Akaike's Information Criterion (AIC) and BIC but used BIC for decision on number of classes) and classification entropy. Individuals were allocated into classes based on the largest probability (maximum a posteriori, MAP).

Once meaningful and reliable subtypes were identified based on symptom scales, we included predictors of latent classes as auxiliary variables in the mixture models (using a 3-step-approach (http://www.statmodel.com/download/webnotes/webnote15.pdf,Asparouhov and Muthén, 2014)) to assess the distribution and prevalence of comorbidities (and neurocognitive functions) across subtypes. We considered the following measures (disorders) as predictors of latent classes: Emotion Regulation Questionnaire - Reappraisal and Suppression subscales (Gross and John, 2003); Distress Tolerance Scale total score (Simons and Gaher, 2005); Adult Sensory Profile: Sensory Sensitivity subscale (Brown, 2002); age; gender; duration of illness; presence of ADHD (threshold for probable caseness on the Adult ADHD Self-Report Scale Screener (Kessler et al., 2005)), presence of obsessive compulsive disorder (OCD) (MINI diagnosis), family history of alcohol or substance use disorder, family history of OCD, psychosocial disability (Sheehan Disability Scale) (Sheehan et al., 1996), Short Mood and Feelings Questionnaire (total scores, depression) (Angold et al., 1995), perfectionism (Frost perfectionism scale total score) (Frost et al., 1990); Barratt Impulsivity Scale (BIS-11, total score) (Patton et al., 1995), extra-dimensional set-shifting errors (CANTAB IED) (Owen et al., 1991), and stop-signal reaction times (CANTAB SSRT) (Aron et al., 2007). The above-mentioned family history assessments were made in first-degree relatives by the probands; relatives were not interviewed. In each case, symptoms were differentiated from an actual diagnosis made by a treatment provider, and for purposes of this study, a diagnosis was only entered as positive if the family member had been formally diagnosed.

These predictors of interest were added separately, one at a time and the corresponding p-values were corrected for multiple testing using the Holm-Bonferroni method. Mixture modeling (including models with predictors) was conducted using MPlus 8.4 (Muthén and Muthén, 2016), and data were further processed in R (R Core Team, 2002).

## 3 Results

### 3.1 Sample characteristics

The final sample included 279 participants (221 with a BFRB and 58 healthy controls), of which 100 had TTM (83.0% female; mean age = 30.8 ± 9.7), 81 had SPD (88.9% female; mean age = 32.4 ± 11.3), and 40 had both TTM and SPD (87.5% female; mean age = 27.0 ± 8.1)(i.e. met full DSM-5 diagnostic criteria for both disorders). Of the 58 healthy controls, 45 (77.6%) were female and the mean age was 29.2 ± 11.2 years. Demographic data of those participants with BFRBs at each site is presented in Table 1.

**Table 1:** Demographic data for the 279 adults participants based on study site.

	University of Chicago (n = 93)	University of California, Los Angeles (n = 87)	Massachusetts General Hospital/Harvard Medical School (n = 84)	Stellenbosch University (n = 15)
Females, n (%)	77 [83.7%]	67 [77.9%]	65 [77.4%]	14 [93.3%]
Mean Age (SD)	30.1 (8.5)	29.8 (10.4)	30.5 (11.4)	34.9 (15.8)
Trichotillomania, n (%)	37 [40.0%]	33 [37.9%]	17 [20.4%]	13 [86.7%]
Skin Picking Disorder, n (%)	32 [34.4%]	17 [19.5%]	32 [38.1%]	0 [0%]
Comorbid trichotillomania plus skin picking disorder, n (%)	10 [10.8%]	16 [18.4%]	12 [14.3%]	2 [13.3%]
Controls, n (%)	14 [15.1%]	21 [24.1%]	23 [27.4%]	0 [0%]

### 3.2 Identification of subtypes

Fit parameters for the models from mixture models are outlined in Tables 2a and Table 2b (for additional statistical details see Supplement). For the TTM subtype analysis, the BIC data suggested that a 4-class model was the best fitting model statistically. Entropy of the final 4-class model was 0.98, indicating excellent class separation (entropy is a measure of reliability of classification and indication of separation of classes, and ranges from 0 to 1, with higher values indicating better class separation and more reliable classification of individuals into corresponding classes). For the SPD subtype analysis, the BIC data suggested that a 3-class model was the best fitting model statistically. Entropy of the final 3-class model was 0.99, indicating excellent class separation.

**Table 2a:** Summary of model fit parameters from Mixture Modeling Analysis. Trichotillomania subtypes based on combined item-level data from MGH-HPS and MIST-A-R.

Title	Observations	Parameters	AIC	BIC	Entropy
1-classes;	279	54	22635	22831	NA
2-classes;	279	96	18043	18392	0.999
3-classes;	279	138	17540	18042	0.976
4-classes;	279	180	17213	17868	0.980
5-classes;	279	222	17104	17911	0.980
6-classes;	279	264	17068	18027	0.975

**Table 2b:** Skin Picking Disorder subtypes based on combined item-level data from MIDAS and SPS-R.

Title	Observations	Parameters	AIC	BIC	Entropy
1-classes;	279	54	13761	14048	NA
2-classes;	279	96	9943	10521	0.999
3-classes;	279	138	9478	10347	0.985
4-classes;	279	180	9310	10469	0.986
5-classes;	279	222	9249	10699	0.989
6-classes;	279	264	9261	11002	0.990

Abbreviations: MGH-HPS = Massachusetts General Hospital Hair Pulling Scale; MIST-A-R = Milwaukee Inventory for Subtypes of Trichotillomania-Adult-Revised; MIDAS = Milwaukee Inventory for the Dimensions of Adult Skin Picking; SPS-R=Skin Picking Scale-Revised.

**Fig. 1:**
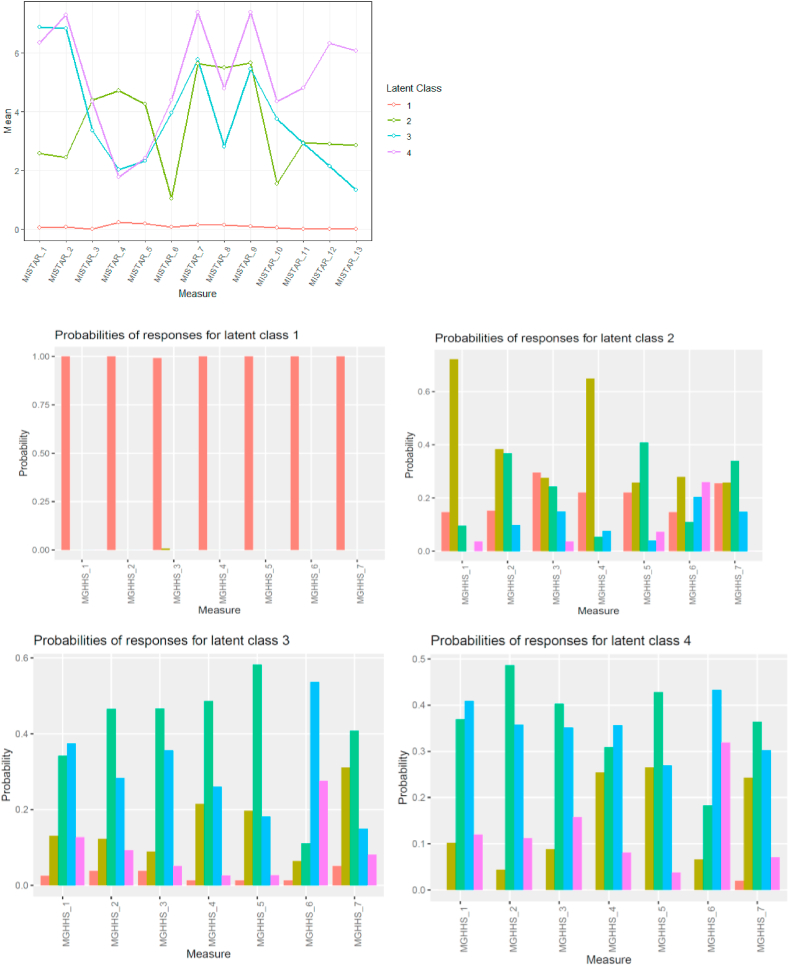
Profiles of latent subtypes on the MIST-A-R (Top graph; all latent subtypes combined), and MGH-HPS (Bottom graphs for each individual latent subtype).

**Fig. 2:**
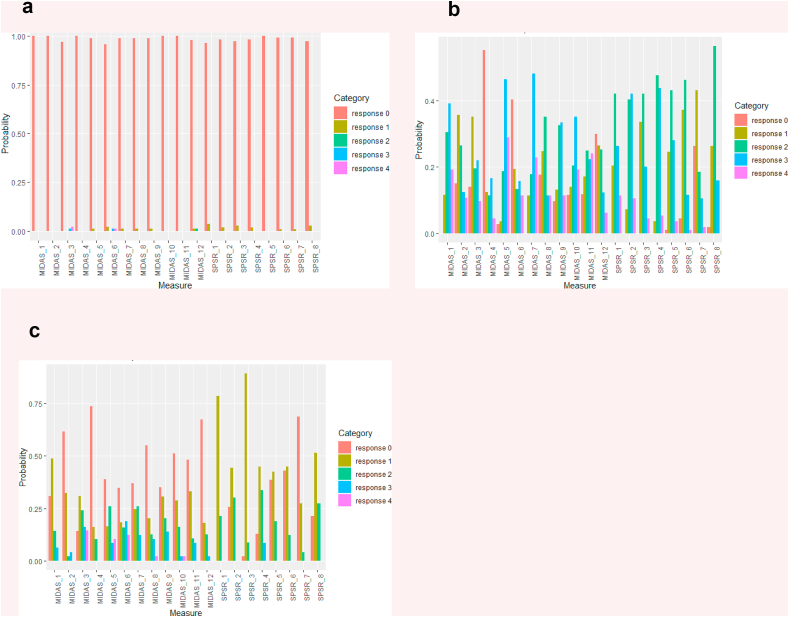
Profiles of latent subtypes on the MIDAS (Milwaukee Inventory for the Dimensions of Skin Picking) and (SPS-R) Skin Picking Scale Revised.

Fig. 1, Fig. 2 show the profiles of the identified classes. In terms of TTM, Class 1 (n = 121) had no TTM cases, and was defined as the TTM-absent group. There were essentially 3 latent subtypes of TTM, which we refer to as Subtypes 1, 2, and 3. Subtype 1 (n = 27) is characterized by the following: highly focused pulling, but low frequency and intensity of urges to pull, and lower frequency of pulling behavior. Subtype 2 (n = 81) is characterized by automatic pulling with fairly low urges to pull but report pulling due to emotional triggers. The unique characteristics of Subtype 3 (n = 50) are that they pull largely to control unpleasant feelings and feel generally unable to resist their pulling.

In terms of SPD, Class 1 (n = 115) had no SPD cases, and was defined as the SPD-absent group. There were essentially 2 latent subtypes of SPD, which we refer to as Subtypes 1 and 2. Subtype 1 (n = 112) is characterized by strong and frequent urges to pick, picking both from negative emotions as well as automatic picking, and reporting little control. Subtype 2 (n = 52) comprises a group of people with milder SPD symptoms as they report low urges to pick, not picking due to emotional issues, spending less time picking, and reporting little distress or impact from the picking.

### 3.3 Differences between the latent class subtypes on variables of interest

Each latent subtype of TTM was compared on a number of variables to characterize the subtypes clinically (see Table 3). This was also performed on each latent subtype of SPD (see Table 4).

**Table 3:** Differences in variables of interest across Trichotillomania (TTM) subtypes.

Variable	Class 1 TTM-Absent/Controls (n = 121)	TTM Subtype 1 (n = 27)	TTM Subtype 2 (n = 81)	TTM Subtype 3 (n = 50)	Statistic – Overall	Post hoc tests
Demographics and Clinical Measures						
**Age, yrs**	30.588 (0.95)	33.136 (2.288)	29.186 (1.041)	30.37 (1.452)	Chi-square = 2.769; df = 3 p = 0.429 (corrected p value = 1)	
**Sex, proportion females**	0.846	0.885	0.815	80.1	Chi-square = 0.857; df = 3; p = .836 (corrected p value = 1)	
**Duration of illness, yrs**	18.27 (1.426)	19.258 (2.33)	17.119 (1.211)	17.271 (1.592)	Chi-square = 0.909; df = 3; p = 0.823 (corrected p value = 1)	
**ADHD symptoms (Adult ADHD Self-Report Scale Screener total scores)**	1.359 (0.149)	1.652 (0.339)	2.232 (0.215)	2.302 (0.268)	Chi-square = 16.244; df = 3; p = .001 (corrected p value = .013)	Subtype 2 compared to controls (p = .001) and Subtype 3 compared to controls (p = .003)
**Impairment due to BFRB (Sheehan Disability Scale total score)**	4.389 (0.515)	7.54 (1.434)	7.059 (0.789)	10.776 (1.159)	Chi-square = 29.705; df = 3; p < .001 (corrected p value < .001)	Subtype 1 compared to controls (p = .039) Subtype 2 compared to controls (p = .005) Subtype 3 compared to controls (p < .001) Subtype 3 compared to subtype 2 (p = .008)
**Short Mood and Feelings Questionnaire**	3.599 (0.386)	6.684 (1.104)	5.111 (0.599)	7.783 (0.872)	Chi-square = 24.298; df = 3; p < .001 (corrected p value < .001)	Subtype 1 compared to controls (p = .008) Subtype 2 compared to controls (p = .034) Subtype 3 compared to controls (p < .001) Subtype 3 compared to subtype 2 (p = .012)
**The Emotion Regulation Questionnaire – Reappraisal Subscale**	28.112 (0.652)	26.062 (1.471)	28.068 (0.776)	25.337 (1.118)	Chi-square = 6.117; df = 3; p = .106 (corrected p value = 1)	
**Suppression Subscale**	12.891 (0.473)	14.846 (1.05))	13.197 (0.574)	14.283 (0.746)	Chi-square = 4.577; df = 3; p = .206 (corrected p value = 1)	
**Distress Tolerance Scale total score**	56.685 (1.129)	51.615 (2.816)	52.919 (1.46)	43.629 (1.852)	Chi-square = 36.509; df = 3; p < .001 (corrected p value < .001)	Subtype 3 compared to controls (p < .001) compared to Subtype 1 (p = .018) and compared to Subtype 2 (p < .001) Subtype 2 compared to controls (p = .041)
**Adult sensory profile: Sensory Sensitivity subscale**	30.749 (0.701)	37.684 (1.808)	34.189 (0.966)	36.063 (1.278)	Chi-square = 24.303; df = 3; p < .001 (corrected p value < .001)	Subtype 1 compared to controls (p < .001) Subtype 2 compared to controls (p = .004) Subtype 3 compared to controls (p < .001)
**Perfectionism (Frost Perfectionism in Adults total score)**	80.011 (1.961)	80.032 (4.274)	84.746 (2.464)	95.562 (3.047)	Chi-square = 19.468; df = 3; p < .001 (corrected p value = .003)	Subtype 3 compared to controls (p < .001) compared to Subtype 1 (p = .003) and compared to Subtype 2 (p = .006)
**Comorbidity**						
**ADHD diagnosis, proportion meeting diagnostic criteria**	0.017	0	0	0.061	Chi-square = 5.082; df = 3; p = .166 (corrected p value = 1)	
**Obsessive Compulsive Disorder (OCD) diagnosis, proportion with diagnosis**	0.017	0.074	0.037	0.141	Chi-square = 6.890; df = 3; p = .075 (corrected p value = .830)	
**Family History**						
**Family History of OCD, proportion with**	0.0125	0	0.037	0.020	Chi-square = 7.198; df = 3; p = .066 (corrected p value = .790)	
**Family history of Alcohol use disorder, proportion with**	0.182	0.146	0.178	0.213	Chi-square = 0.422; df = 3; p = .936 (corrected p value = 1)	
**Family history of Substance use disorder, proportion with**	0.074	0.073	0.099	0.142	Chi-square = 1.461; df = 3; p = .691 (corrected p value = 1)	
**Cognitive Measures**						
**Barratt Impulsivity Scale total**	56.12 (1.103)	58.593 (2.398)	59.935 (1.266)	64.282 (1.613)	Chi-square = 18.097; df = 3; p < .001 (corrected p value = .006)	Subtype 3 compared to controls (p < .001), compared to Subtype 1 (p = .049) and compared to subtype 2 (p = .034) Subtype 2 compared to controls (p = 0.023)
**Extra-dimensional set-shifting errors**	9.491 (0.963)	12.719 (2.32)	9.874 (1.225)	10.18 (1.653)	Chi-square = 1.674; df = 3; p = .643 (corrected p value = 1)	
**Stop-signal reaction times**	207.762 (6.387)	239.64 (30.529)	220.784 (11.386)	247.528 (25.006)	Chi-square = 3.801; df = 3; p = 1 (corrected p value = 1)	

Values are Mean (±SE) unless stated otherwise; only significant results are displayed under post-hoc tests.

**Table 4:** Differences in variables of interest across skin picking disorder (SPD) subtypes.

Variable	Class 1 SPD-Absent/Controls (n = 115)	SPD Subtype 1 (n = 112)	SPD Subtype 2 (n = 52)	Statistic – Overall	Post hoc tests
Demographic and Clinical Measures					
**Age, yrs**	29.78 (0.937)	30.244 (0.967)	32.1 (1.644)	Chi-square = 1.51; df = 2; p = 0.47 (corrected p value = 1)	
**Sex, proportion female**	0.816	0.840	0.856	Chi-square = 0.460; df = 2; P = 0.795 (corrected p value = 1)	
**Duration of illness, yrs**	17.557 (1.452)	17.821 (1.103)	18.095 (1.7)	Chi-square = 0.058; df = 2; P = 0.971 (corrected p value = 1)	
**ADHD symptoms (Adult ADHD Self-Report Scale Screener total scores)**	1.149 (0.142)	2.478 (0.182)	1.834 (0.257)	Chi-square = 33.606; df = 2; p < .001 (corrected p value < .001)	Subtype 1 compared to controls (p < .001) and compared to subtype 2 (p = .041) Subtype 2 compared to controls (p = .02)
**Impairment due to BFRB (Sheehan Disability Scale total score)**	3.861 (0.482)	9.817 (0.741)	5.706 (0.932)	Chi-square = 45.364; df = 2; p < .001 (corrected p value < .001)	Subtype 1 compared to controls (p < .001) and compared to Subtype 2 (p = .001)
**Short Mood and Feelings Questionnaire**	3.345 (0.386)	7.221 (0.578)	4.651 (0.696)	Chi-square = 31.116; df = 2; p < .001 (corrected p value < .001)	Subtype 1 compared to controls (p < .001) and compared to Subtype 2 (p = .005)
**The Emotion Regulation Questionnaire – Reappraisal Subscale**	28.727 (0.672)	26.158 (0.707)	26.748 (1.052)	Chi-square = 7.359; df = 2; p = .025 (corrected p value = .303)	
**Suppression Subscales**	12.82 (0.495)	13.742 (0.5)	13.846 (0.745)	Chi-square = 2.191; df = 2; p = .334 (corrected p value = 1)	
**Distress Tolerance Scale total score**	56.37 (1.205)	48.566 (1.334)	51.701 (1.932)	Chi-square = 19.135; df = 2; p < .001 (corrected p value < .001)	Subtype 1 compared to controls (p < .001) Subtype 2 compared to controls (p = .04)
**Adult Sensory Profile: Sensory Sensitivity subscale**	30.043 (0.704)	36.933 (0.816)	32.874 (1.22)	Chi-square = 40.862 df = 2; p < .001 (corrected p value < .001)	Subtype 1 compared to controls (p < .001) and compared to Subtype 2 (p = .006) Subtype 2 compared to controls (p = .044)
**Perfectionism (Frost Perfectionism in Adults total score)**	78.104 (2.027)	90.544 (2.132)	83.618 (3.028)	Chi-square = 17.894 df = 2; p < .001 (corrected p value = .002)	Subtype 1 compared to controls (p < .001)
**Comorbidity**					
**ADHD diagnosis, proportion meeting diagnostic criteria**	0	0.044	0	Chi-square = 5.193 df = 2; p = .075 (corrected p value = .745)	
**Obsessive Compulsive Disorder (OCD) diagnosis, proportion with diagnosis**	0.035	0.080	0.019	Chi-square = 3.588 df = 2; p = .166 (corrected p value = 1)	
**Family History**					
**Family History of OCD, proportion with**	0.009	0.035	0.039	Chi-square = 2.685 df = 2; p = .261 (corrected p value = 1)	
**Family history of Alcohol use disorder, proportion with**	0.122	0.248	0.196	Chi-square = 6.387 df = 2; p = .041 (corrected p value = .451)	
**Family history of Substance use disorder, proportion with**	0.061	0.124	0.098	Chi-square = 2.832; df = 3; p = .243 (corrected p value = 1)	
**Cognitive Measures**					
**Barratt Impulsivity Scale total**	56.773 (1.139)	61.51 (1.08)	57.582 (1.641)	Chi-square = 9.96 df = 2; p = 0.007 (corrected p value = .089)	
**Extra-dimensional set-shifting errors**	10.674 (1.044)	9.063 (0.999)	10.46 (1.549)	Chi-square = 1.377 df = 2; p = .502 (corrected p value = 1)	
**Stop-signal reaction times**	232.344 (12.414)	218.942 (9.266)	200.741 (8.157)	Chi-square = 5.094 df = 2; p = .078 (corrected p value = .745)	

In terms of the TTM subtypes, Subtype 1 scored highest in sensory sensitivity and had significantly elevated scores compared to controls on impairment and mood symptoms (Table 3). Subtype 2 is notable for some of the same problems of Subtype 1 (mood and impairment issues) but also ADHD symptoms and general impulsivity as seen on the BIS-11. Subtype 3 is perhaps the most striking latent class as it has the same symptoms of the other subtypes (but to a greater degree of impairment and mood symptoms) and additionally scored high on perfectionism, had less distress tolerance, and scored highest on impulsivity (Table 3).

In terms of the SPD subtypes, Subtype 1 had significantly more ADHD symptoms, co-occurring ADHD diagnoses, more impairment and more mood symptoms than the controls or the other latent class (Table 4). Unique to this subtype was high levels of perfectionism and less distress tolerance. Subtype 2 reported some ADHD symptoms relative to controls, some problems with sensory sensitivity relative to controls, poor distress tolerance, but impairment at the same level of controls and no problems with perfectionism.

## 4 Discussion

To our knowledge, this is the largest phenotyping study of BFRBs using multimodal, gold standard assessment tools. This paper makes several important contributions to our understanding of BFRBs. First, instead of being simply homogeneous disorders, mixture modelling (MM) indicated that both TTM and SPD are comprised of separate subtypes, three in the case of TTM and two for SPD. Second, there are unique clinical characteristics of the various subtypes that potentially could be targets for treatment.

### 4.1 Differences between the TTM subtypes

Our results suggest that TTM has three subtypes with unique clinical presentations. Of the three subtypes of TTM, Subtype 1 (which we refer to as “sensory sensitive pullers”) is characterized by highly focused pulling, but urges to pull are generally infrequent and of low intensity. Thus this group has a somewhat lower frequency of pulling behavior. A person in this subtype scores high on measures of sensory sensitivity, is moderately impaired by their pulling, and reports moderate mood symptoms.

A person in Subtype 2 (which we refer to as “low awareness pullers”), the most common subtype (54.2% of TTM participants), reports more automatic pulling, and more pulling due to emotional triggers, with fairly low urges to pull. This person may report some impairment and some mood issues but unlike Subtype 1, they may present with some ADHD symptoms and higher levels of overall impulsivity.

A person in Subtype 3 (which we refer to as “impulsive/perfectionist pullers”) may present with the most unique characteristics of the three groups. These are generally people who pull to control unpleasant feelings and feel generally unable to resist their pulling. They report a greater degree of impairment and mood symptoms, and less distress tolerance than controls or the other subtypes of TTM. They are simultaneously more likely to score higher on measures of perfectionism than the other subtypes, and also score very high on measures of overall impulsivity.

### 4.2 Differences between the SPD subtypes

The two SPD subtypes also exhibited clinical differences from each other. Of the two subtypes of SPD, Subtype 1 (which we refer to “emotional/reward pickers”) which represents the majority of people with SPD is characterized by strong and frequent urges to pick, picking from negative emotions as well as automatic picking, and reporting little control. People in this subtype score high on measures of ADHD and report high levels of perfectionism.

Subtype 2 (which we refer to as “functional pickers”) reflects a group of people with fairly mild SPD, with lower urges to pick, and overall little distress or impact from the picking. Interestingly, these people report some problems with sensory sensitivity and poor distress tolerance. They report minimal impairment due to their picking and do not present problems with emotional dysregulation, perfectionism or impulsivity.

The subtypes of SPD are a bit more problematic than those for TTM. Are these two subtypes truly categorically different, or, might they simply be different ends of a continuum? Considering the move toward dimensional conceptualizations and the lack of robust distinguishing characteristics here, these two subtypes may be reflective of a mild vs more moderate-severe symptomatology, rather than “subtypes”. Future research should examine whether these "subtypes" change over time with treatment as their symptoms improve.

### 4.3 Clinical relevance

One of the possible benefits of understanding the complexity of BFRBs is that treatment can be better tailored. In terms of psychosocial treatments, there are several options that have shown some benefit for BFRBs. Habit reversal training, alone or enhanced with dialectical behavior therapy, or acceptance and commitment therapy, and comprehensive model for behavioral treatment, to name a few, are all associated with some benefit for BFRBs (Falkenstein et al., 2016; Woods and Houghton, 2016). To a lesser extent (in terms of their being fewer studies evaluating such treatments), but with a similar limited efficacy, pharmacotherapy (e.g., clomipramine, olanzapine, and N-acetyl cysteine) has shown some benefit for BFRBs as well (Rothbart et al., 2013). The question of whether there is a subgroup of BFRB which preferentially benefits from a particular therapy or medication, however, has not been answered. Instead of considering the BFRB the therapeutic target, possibly treating these comorbidity symptoms or other differences could reduce BFRB severity.

Based on these data, one could perhaps imagine formulating different treatment plans based on the subgroups of BFRB. For example, those individuals who fit Subtypes 1 and 2 might benefit from learning to increase awareness through either habit reversal training or awareness enhancement devices, whereas those individuals with TTM who fit into Subtype 3 might benefit from psychotherapy or pharmacotherapy focusing on emotional dysregulation. Similarly, those with SPD who fit into Subtype 1 may benefit from interventions that focus on enhancing coping skills/resilience, ADHD, mood or personality (perfectionism), and perhaps may benefit from a medication targeting mood or perfectionism for their BFRB.

Neurocognitive deficits have been demonstrated in patients with TTM and SPD in the literature, compared to controls. Here, the classes did not differ overall in terms of set-shifting or response inhibition performance. It is important to note that, due to the statistical methods used, the control group for the TTM subtypes included SPD cases; and that the control group for the SPD subtypes included TTM cases (as people with picking would answer “no” to all questions about pulling and vice versa). Hence, failure to detect group differences on cognition does not indicate that TTM and SPD as disorders are free from such deficits; rather, just that they are not detectable when groups are defined using the current statistical methodology. It would be valuable in future work to explore cognition in more detail, including in comparison to healthy control reference group(s); and to examine whether TTM/SPD subtypes can be identified based on cognition and imaging markers. At the same time, group differences were found in terms of Barratt Impulsivity Scale scores. This may suggest that self-report measures may be more sensitive to differences in such latent subtypes than these neurocognitive tasks (prior work indicates self-report measures may link more directly with psychopathologies than cognitive tests (Eisenberg et al., 2019).

### 4.4 Limitations

Though this is the largest analysis of potential BFRB subtypes to date, and one of very few studies using mixture modeling in these disorders, several limitations should be considered. The identification of differences between classes could be viewed as conservative, since we first conducted statistical tests to determine if classes differed overall; whereas some classes might be expected to be similar rather than different on a given measure. Given the small sample of comorbid TTM and SPD participants, as well as the fact that some participants had subclinical symptoms of the other disorder, this may complicate our interpretation of these findings. Further, our sample size is arguably small to estimate our mixture models, especially those with larger number of classes. However, we did not experienced symptoms of estimation problems (e.g. large standard errors or unstable solutions) for our final models. Finally, SPD classes, being mainly severity clusters may suggest that the disorder is rather dimensional rather than categorical. More research is needed to investigate this issue.

## 5 Conclusions

Although subtype characteristics have been discussed in the literature and are identified in the course of clinical interviews or as part of the functional analysis at the outset of behavioral treatment, this study is the first to identify more definitely distinct classes of TTM and SPD, using mixture modeling, in a large sample of patients with BFRBs and controls. It also highlights aspects of subtyping that over the years may have been discussed but these data would not support as meaningful (i.e. where the person picks/pulls from; early or late onset, etc). Instead of those numerous possible subtypes in the previous literature, we found evidence for three distinct classes of TTM cases; and arguably two distinct classes of SPD cases. These classes differed remarkably on clinical characteristics and this information may be useful in future to help direct tailored treatments and for further work into discerning the biological underpinnings of these under-studied disorders.

## Funding/support

This study was funded by the Body-Focused Precision Medicine Initiative granted by The TLC Foundation for Body-Focused Repetitive Behaviors to 10.13039/100007234University of Chicago (Dr. Grant), 10.13039/100005294Massachusetts General Hospital (10.13039/100005294MGH)/Harvard (Dr. Keuthen), and University of California, Los Angeles (UCLA) (Dr. Piacentini). The TLC Foundation for Body-Focused Repetitive Behaviors had no role in the design and conduct of the study; collection, management, analysis, and interpretation of the data; preparation, review, or approval of the manuscript; and decision to submit the manuscript for publication. This study was completed with support from the REDCap project at the 10.13039/100007234University of Chicago, which is hosted and managed by the Center for Research Informatics and funded by the Biological Sciences Division and by the Institute for Translational Medicine, 10.13039/100016220CTSA grant number UL1 TR000430 from the 10.13039/100000002National Institutes of Health. Dr. Chamberlain's research work is funded by a 10.13039/100010269Wellcome Trust Clinical Fellowship (United Kingdom; reference 110049/Z/15/Z).

## Author Contributions

Jon E. Grant, Tara Peris, Emily Rickets, Christine Lochner, Dan, J. Stein, Jan Stochl, Samuel R. Chamberlain, Jeremiah Scharf, Darin Dougherty, Doug Woods, John Piacentini, Nancy J. Keuthen, Conceptualization, Funding acquisition, Formal analysis, Data curation, Writing - original draft, Investigation, all made substantial contributions to the conception or design of the work as well as the acquisition, analysis, or interpretation of data; they all aided in drafting the work, gave final approval of the version to be published; and agree to be accountable for all aspects of the work in ensuring that questions related to the accuracy or integrity of any part of the work are appropriately investigated and resolved

## Declaration of Competing interest

Dr. Grant has received research grants from Biohaven, Promentis, and Otsuka Pharmaceuticals. Dr. Grant receives yearly compensation from Springer Publishing for acting as Editor-in-Chief of the Journal of Gambling Studies and has received royalties from Oxford University Press, American Psychiatric Publishing, Inc., Norton Press, and McGraw Hill. Dr. Stein and Dr. Lochner are supported by the SAMRC Unit on Risk & Resilience in Mental Disorders. Dr. Stein has received research grants and/or consultancy honoria from Lundbeck and Sun. Dr. Chamberlain consults for Ieso and Promentis; and receives stipends from Elsevier for editorial journal work. The other authors report no conflicts. Dr. Dougherty receives research support and honoraria from Medtronic, Inc. Dr. Woods has received royalties from Oxford University Press and Springer Press. Dr. Piacentini has received research grants from NIMH, the Tourette Association of America, and Pfizer. He receives travel support and honoraria from the Tourette Association of America and the International OCD Foundation and book royalties from Guilford Publications and Oxford University Press. Dr Keuthen has received prior research grants from The TLC Foundation for Body-Focused Repetitive Behaviors and royalties from New Harbinger, Inc. The remaining authors have nothing to disclose.
