# Supplementary material for: Identifying subtypes of trichotillomania (hair pulling disorder) and excoriation (skin picking) disorder using mixture modeling in a multicenter sample
Source: J Psychiatr Res. 2021 May;137:603–12. doi: 10.1016/j.jpsychires.2020.11.001 (PMC7610704; doi:10.1016/j.jpsychires.2020.11.001)
Supplement: Multimedia component 1 [file mmc1.docx]

Supplement

Supplement 1. All measures used in the study

| **Adult Battery** |
| --- |
|  |
| *Demographic Form:* Multiple choice self-report of age, ethnicity, gender, race, educational attainment, marital status, family income, educational attainment, SES, and patient and family psychiatric/medical treatment history.  [10 minutes] |
| Family History of Psychiatric Diagnoses: Coded self-report of family history of psychiatric diagnoses.  [4 minutes] |
| *Mini International Neuropsychiatric Interview 7.0 (MINI 7.0; Lecrubier et al., 1997):* A brief structured clinician-administered diagnostic interview assessing DSM-IV psychiatric disorders in adults.  [35 minutes] |
| *Suicidal Behaviors Questionnaire (SBQ; Linehan, 1981):* The 11-item self-report checklist portion of the SBQ will be used. It assesses suicidal and self-harm behaviors occurring in the past year will be used. It may be completed as a self-report measure but must be reviewed by the independent evaluator right after completion. This measure cannot be placed on REDCAP.  [3 minutes] |
|  |
| *Massachusetts General Hospital Hair Pulling Scale (MGH-HPS; Keuthen et al., 1995):* Self-report measure (7-item likert scale) assesses urge frequency, intensity, and controllability, hair pulling frequency, resistance, and controllability, and associated distress during the prior week. The total score ranges from 0 to 28, with higher scores indicating greater severity and lower scores indicating decreased severity.  [2 minutes] |
| *Hair Pulling Urge Scale:* A brief self-report measure of urge to engage in hair pulling.  [1 minute] |
| *Milwaukee Inventory for Subtypes of Trichotillomania-Adult Version (MIST-A; Flessner et al, 2008):* A 15-item self-report measure, with a 10-item focused pulling scale and a 5-item automatic pulling scale, yielding independent total scores.  [3 minutes] |
| *Skin Picking Scale-Revised (SPS-R):* An 8-item self-report measure of skin picking severity in the past week.  [2 minutes] |
| *Skin Picking Urge Scale:* A brief self-report measure of urge to engage in skin picking.  [1 minute] |
| *Milwaukee Inventory for the Dimensions of Skin Picking (MIDAS; Walther, Flessner, Conelea, & Woods, 2009):* A 12-item self-report measure assessing the degree to which skin picking is automatic (occurring without awareness), and focused.  [2 minutes] |
| *Yale-Brown Obsessive Compulsive Scale (Y-BOCS; Goodman et al., 1989):* A clinician-rated interview assessing current or past obsessive thoughts and compulsive behaviors. Both the severity scale and symptom checklist will be used.  [15 minutes] |
| *Adult Tic Questionnaire (ATQ; unpublished):* The ATQ is an adult rating scale identical in structure to the PTQ, assessing tic frequency and intensity. It feature subtotals for motor and vocal tics, with an overall total score.  [5 minutes] |
| *Premonitory Urge for Tics Scale (PUTS; Woods, Piacentini, Himle, & Chang, 2005):* The PUTS is a brief 9-item measure of premonitory tic sensations validated in individuals ages 8 to 16.  [1 minute] |
| *Mood and Feelings Questionnaire – Short Version (MFQ-Short; Angold et al., 1995):* A brief 13-item measure of depressive symptoms in adults.  [2 minutes] |
| *Depression Anxiety Stress Scale – 21 (DASS-21; Lovibond & Lovibond, 1995):* A 21-item measure of depression, anxiety, and stress symptoms in adults in the past week. It yields total scores for each of the three domains.  [3 minutes] |
| *Adult ADHD Self-Report Scale (ASRS; Kessler et al., 2005):* A brief 18-item self-report measure of ADHD symptoms based on DSM-IV diagnostic criteria.  [3 minutes] |
| *Body Esteem Scale for Adolescents and Adults (BESAA; Mendelson et al., 2001):*  A self-report measure for ages 16 and older assessing body esteem in adolescents and adults. It includes 3 subscales: appearance, weight, and attribution.  [3 minutes] |
| *Quality of Life Inventory (Frisch, 1988):* A 17-item measure of life satisfaction. Items are rated in terms of their significance to the rater, and the rater’s satisfaction with the area. Higher scores reflect greater satisfaction.  [2 minutes] |
| *Sheehan Disability Scale (Sheehan, Harnett-Sheehan, & Raj, 1996):* A brief self-report scale, including 3 items assessing disability and functional impairment at work/school, social life/leisure activities, and family life/home responsibilities. Each item includes a 10-point likert scale with anchors ranging from “not at all” to “extremely”.  [1 minute] |
| *Pittsburgh Sleep Quality Index (PSQI; Buysse, Reynolds, Monk, Berman, & Kupfer, 1989):* A 19-item self-report measure of sleep quality over the past month with component scores for sleep quality, sleep latency, sleep duration, habitual sleep efficiency, sleep disturbance, use of sleeping medication, and daytime dysfunction; and a global sleep disturbance score.  [3 minutes] |
| *Munich Chronotype Questionnaire (MCTQ; Allebrandt & Roenneberg, 2008):* A 13-item self-report measure of circadian preference on scheduled and unscheduled days for individuals ages 6 to 65.  [2 minutes] |
| **Affect Regulation** |
| *Emotion Regulation Questionnaire (Gross & John, 2003):* The ERQ is a 10-item measure of use of both cognitive reappraisal and expressive suppression emotion regulation strategies. Items are rated on a 7-point likert scale with higher scores indicative of more frequent use of these emotion regulation strategies.  [1 minute] |
| *Distress Tolerance Scale (Simons & Gaher, 2005):* A 20-item self-report measure of ability to tolerate distress. It is based on a model encompassing four areas, including emotional tolerance, appraisal, absorption, and regulation. Items are rated on a 5-point likert scale. Increased ratings signify greater levels of distress tolerance.  [3 minutes] |
| *Anxiety Sensitivity Index - 3 (ASI-3; Taylor et al., 2007):* The ASI-3 is an 18-item measure of anxiety sensitivity across three domains: somatic, social, and cognitive concerns. Items are rated on a 5-point likert scale from 0 (very little) to 4 (very much).  [3 minutes] |
| *Acceptance and Action Questionnaire – Trichotillomania (AAQ-TTM):* A 10-item self-report measure of psychological inflexibility related to hair pulling urges. It includes a 7-point likert scale, with higher scores indicative of greater psychological flexibility.  [1 minute] |
| *Family Environment Scale (FES; Moos & Moos, 1986):* A 90-item self-report measure with 10 subscales spanning 3 core domains. Domains include Interpersonal Relationship (Cohesion, Expressiveness, and Conflict); Personal Growth (independence, achievement orientation, intellectual-cultural orientation, active-recreational orientation, and moral-religious emphasis; and System Maintenance (organization, and control). The underlined scales will be used in the present study.  [4 minutes] |
| *Family Adaptability and Cohesion Scale – IV (FACES-IV; Olson, 2011):* A 62-item measure of family member satisfaction with the family system tapping cohesion and flexibility specifically. It features 6 subscales, including balanced cohesion and balanced flexibility; disengaged and enmeshed (Cohesion Dimension), and rigid and chaotic (Flexibility Dimension). This measure is for all family members ages 12 and above.  [8 minutes] |
| **Impulsivity** |
| *Barratt Impulsiveness Scale (BIS-11; Barratt, 1994):* Includes three subscales: attentional impulsivity, motor impulsivity, and non-planning impulsivity.  [4 minutes] |
| *Behavioral Inhibition System/Behavioral Activation System (Carver & White, 1994):* A self-report measure assessing two motivation systems proposed to underlie behavior, including desire to move away from stimuli perceived as aversive (Behavioral Inhibition Scale) and desire to move towards something desirable. The scale includes 4 subscales scores, including: BAS Drive, BAS Fun Seeking, BAS Reward Responsiveness, and BIS.  [3 minutes] |
| *Alcohol Use Disorders Identification Test (AUDIT; Saunders et al., 1993):* A 10-item self-report screening measure that provides a total scores range from 0 to 40.  [2 minutes] |
| *Drug Abuse Screen Test (DAST-10; Cocco & Kate, 1998):* A 10-item screening measure assessing drug abuse (not including alcohol) within the past 12 months. Total scores range from 0 to 10. The scale allows individuals’ degree of problems related to drug abuse to be categorized into low (1-2), moderate (3-5), substantial (6-8), severe (9-10).  [2 minutes] |
| *NEO Five Factor Inventory (NEO-FFI; Costa & McCrae, 1992):* A 60-item self-report measure of personality assessing 5 domains (openness to experience, conscientiousness, extraversion, agreeableness, and neuroticism) based on the five factor personality model.  [8 minutes] |
| *Perceived Stress Scale (PSS-10; Cohen & Williamson, 1988):* A 10-item measure of perceptions of stress in the past month. It includes the frequency with which individuals report stress-related thoughts and feelings.  [2 minutes] |
| *Multidimensional Perfectionism Scale (Frost, Marten, Lahart, & Rosenblate, 1990):* A 35-item questionnaire assessing 6 domains including concern over mistakes, personal standards, parental expectations, parental criticism, doubts about action, and organization.  [4 minutes] |
| *Stop Signal Task (SST; Sahakian & Owen, 1992):* The SST assesses inhibitory control by requiring the subject to press keys that correspond with right and left arrow stimuli, except when an audio tone is played. The task is being used to gauge impulsivity within BFRBs.  [20 minutes] |
| *Intra-Extra Dimensional Set Shift (IED; Sahakian & Owen, 1992):* The IED is a computer task assessing rule acquisition and reversal. Two simple color-filled shapes are presented and the participant learns which one is correct by touching it and receiving feedback. After 6 correct responses, the stimuli and/or rules change, with shifts being intra-dimensional (e.g., selections based on color-filled shapes) at first, but extra-dimensional (e.g., selections based on white lines) later. In order to progress through each stage, participants must make 6 consecutive correct responses. If the participant fails to achieve this criterion after 50 trials at any stage, the task ends. The test is being used to gauge compulsivity in BRFBs.  [7 minutes] |
| *Cambridge Gambling Task (CGT; Sahakian & Owen, 1992):* The CGT is a computer task assessing decision-making and risk-taking. It measures these constructs outside of a learning context. A row of ten boxes are displayed at the top of the screen, with rectangles depicting the words “red” and blue” at the bottom of the screen. Participants select the red or blue box to indicate where a yellow token is hidden. During the gambling phases, participants begin with several points displayed on the screen, and can select a portion of these points, displayed in either rising or falling order, in a second box on the screen, to gamble on their confidence in their judgment regarding the yellow token’s location. The aim is to collect as many points as possible. The orbitofrontal prefrontal cortex is likely implicated in this task. The task is being used to gauge decision making in BFRBs.  [30 minutes] |

**Supplement 2. Tables indicating additional statistical details for the Mixture Model analysis results.**

**Model probabilities for MGH-HPS + MIST**

**AIC**

| **Modnames** | **K** | **AIC** | **Delta_AIC** | **ModelLik** | **AICWt** | **LL** | **Cum.Wt** |
| --- | --- | --- | --- | --- | --- | --- | --- |
| LPA: 8-classes; | 88 | 13661 | 0 | 1 | 1 | -6743 | 1 |
| LPA: 7-classes; | 78 | 13767 | 105.7 | 1.128e-23 | 1.128e-23 | -6805 | 1 |
| LPA: 6-classes; | 68 | 13884 | 222.4 | 5.031e-49 | 5.031e-49 | -6874 | 1 |
| LPA: 5-classes; | 58 | 14006 | 345.2 | 1.108e-75 | 1.108e-75 | -6945 | 1 |
| LPA: 4-classes; | 48 | 14174 | 512.4 | 5.526e-112 | 5.526e-112 | -7039 | 1 |
| LPA: 3-classes; | 38 | 14685 | 1024 | 4.547e-223 | 4.547e-223 | -7305 | 1 |
| LPA: 2-classes; | 28 | 15008 | 1347 | 3.66e-293 | 3.66e-293 | -7476 | 1 |
| LPA: 1-classes; | 18 | 16687 | 3026 | 0 | 0 | -8325 | 1 |

**BIC**

| **Modnames** | **K** | **BIC** | **Delta_BIC** | **ModelLik** | **BICWt** | **LL** | **Cum.Wt** |
| --- | --- | --- | --- | --- | --- | --- | --- |
| LPA: 8-classes; | 88 | 13988 | 0 | 1 | 1 | -6743 | 1 |
| LPA: 7-classes; | 78 | 14057 | 68.51 | 1.33e-15 | 1.33e-15 | -6805 | 1 |
| LPA: 6-classes; | 68 | 14136 | 148.1 | 6.991e-33 | 6.991e-33 | -6874 | 1 |
| LPA: 5-classes; | 58 | 14222 | 233.7 | 1.815e-51 | 1.815e-51 | -6945 | 1 |
| LPA: 4-classes; | 48 | 14352 | 363.7 | 1.067e-79 | 1.067e-79 | -7039 | 1 |
| LPA: 3-classes; | 38 | 14826 | 838.1 | 1.035e-182 | 1.035e-182 | -7305 | 1 |
| LPA: 2-classes; | 28 | 15112 | 1124 | 9.818e-245 | 9.818e-245 | -7476 | 1 |
| LPA: 1-classes; | 18 | 16754 | 2765 | 0 | 0 | -8325 | 1 |

**Technical summary of final model**

| **Mplus.version** | 8.4 |
| --- | --- |
| **Title** | LPA: 4-classes; |
| **AnalysisType** | mixture |
| **DataType** | INDIVIDUAL |
| **Estimator** | MLR |
| **Observations** | 304 |
| **NGroups** | 1 |
| **NDependentVars** | 9 |
| **NIndependentVars** | 0 |
| **NContinuousLatentVars** | 0 |
| **NCategoricalLatentVars** | 1 |
| **Parameters** | 48 |
| **LL** | -7038.77 |
| **LLCorrectionFactor** | 1.2494 |
| **AIC** | 14173.54 |
| **BIC** | 14351.96 |
| **aBIC** | 14199.73 |
| **Entropy** | 0.982 |
| **T11_KM1Starts** | 100 |
| **T11_KM1Final** | 20 |
| **T11_KM1LL** | -7304.552 |
| **T11_VLMR_2xLLDiff** | 531.564 |
| **T11_VLMR_ParamDiff** | 10 |
| **T11_VLMR_Mean** | 52.256 |
| **T11_VLMR_SD** | 67.026 |
| **T11_VLMR_PValue** | 1e-04 |
| **T11_LMR_Value** | 522.426 |
| **T11_LMR_PValue** | 1e-04 |
| **BLRT_RequestedDraws** | Varies |
| **BLRT_KM1AnalysisStarts** | 100 |
| **BLRT_KM1AnalysisFinal** | 20 |
| **BLRT_KM1GenStarts** | 0 |
| **BLRT_KM1GenFinal** | 0 |
| **BLRT_KGenStarts** | 40 |
| **BLRT_KGenFinal** | 8 |
| **BLRT_KM1LL** | -7304.552 |
| **BLRT_2xLLDiff** | 531.564 |
| **BLRT_ParamDiff** | 10 |
| **BLRT_PValue** | 0 |
| **BLRT_SuccessfulDraws** | 5 |
| **AICC** | 14191.99 |
| **Filename** | 4-class.out |

**Model Probabilities for MIDAS + SPS-R**

**AIC**

| **Modnames** | **K** | **AIC** | **Delta_AIC** | **ModelLik** | **AICWt** | **LL** | **Cum.Wt** |
| --- | --- | --- | --- | --- | --- | --- | --- |
| LPA: 5-classes; | 399 | 9220 | 0 | 1 | 0.9873 | -4211 | 0.9873 |
| LPA: 6-classes; | 479 | 9229 | 8.712 | 0.01283 | 0.01267 | -4135 | 1 |
| LPA: 4-classes; | 319 | 9281 | 60.64 | 6.809e-14 | 6.722e-14 | -4321 | 1 |
| LPA: 3-classes; | 239 | 9451 | 230.4 | 9.197e-51 | 9.08e-51 | -4486 | 1 |
| LPA: 2-classes; | 159 | 9922 | 701.4 | 4.911e-153 | 4.849e-153 | -4802 | 1 |
| LPA: 1-classes; | 79 | 13729 | 4508 | 0 | 0 | -6785 | 1 |

**BIC**

| **Modnames** | **K** | **BIC** | **Delta_BIC** | **ModelLik** | **BICWt** | **LL** | **Cum.Wt** |
| --- | --- | --- | --- | --- | --- | --- | --- |
| LPA: 3-classes; | 239 | 10319 | 0 | 1 | 1 | -4486 | 1 |
| LPA: 4-classes; | 319 | 10439 | 120.7 | 6.149e-27 | 6.149e-27 | -4321 | 1 |
| LPA: 2-classes; | 159 | 10499 | 180.5 | 6.429e-40 | 6.429e-40 | -4802 | 1 |
| LPA: 5-classes; | 399 | 10669 | 350.6 | 7.502e-77 | 7.502e-77 | -4211 | 1 |
| LPA: 6-classes; | 479 | 10968 | 649.8 | 7.994e-142 | 7.994e-142 | -4135 | 1 |
| LPA: 1-classes; | 79 | 14016 | 3697 | 0 | 0 | -6785 | 1 |

**Technical summary of final model**

| **Mplus.version** | 8.4 |
| --- | --- |
| **Title** | LPA: 3-classes; |
| **AnalysisType** | mixture |
| **DataType** | INDIVIDUAL |
| **Estimator** | MLR |
| **Observations** | 279 |
| **NGroups** | 1 |
| **NDependentVars** | 20 |
| **NIndependentVars** | 0 |
| **NContinuousLatentVars** | 0 |
| **NCategoricalLatentVars** | 1 |
| **Parameters** | 239 |
| **LL** | -4486.327 |
| **LLCorrectionFactor** | 1.0026 |
| **AIC** | 9450.654 |
| **BIC** | 10318.51 |
| **aBIC** | 9560.666 |
| **Entropy** | 0.993 |
| **T11_KM1Starts** | 100 |
| **T11_KM1Final** | 20 |
| **T11_KM1LL** | -4801.818 |
| **T11_VLMR_2xLLDiff** | 630.982 |
| **T11_VLMR_ParamDiff** | 80 |
| **T11_VLMR_PValue** | 0.7605 |
| **T11_LMR_Value** | 629.584 |
| **T11_LMR_PValue** | 0.7605 |
| **BLRT_RequestedDraws** | Varies |
| **BLRT_KM1AnalysisStarts** | 100 |
| **BLRT_KM1AnalysisFinal** | 20 |
| **BLRT_KM1GenStarts** | 0 |
| **BLRT_KM1GenFinal** | 0 |
| **BLRT_KGenStarts** | 40 |
| **BLRT_KGenFinal** | 8 |
| **BLRT_KM1LL** | -4801.818 |
| **BLRT_2xLLDiff** | 630.982 |
| **BLRT_ParamDiff** | 80 |
| **BLRT_PValue** | 0 |
| **BLRT_SuccessfulDraws** | 5 |
| **AICC** | 12392.19 |
| **Filename** | 3-class.out |

**Subtypes based on combined data from mgh-hps and mist-a-r (no controls)**

**Table of model fit**

| **Title** | **Observations** | **Parameters** | **LL** | **AIC** | **BIC** | **aBIC** |
| --- | --- | --- | --- | --- | --- | --- |
| LPA: 1-classes; | 222 | 54 | -9136 | 18380 | 18564 | 18393 |
| LPA: 2-classes; | 222 | 96 | -7661 | 15513 | 15840 | 15536 |
| LPA: 3-classes; | 222 | 138 | -7423 | 15122 | 15591 | 15154 |
| LPA: 4-classes; | 222 | 180 | -7264 | 14888 | 15501 | 14930 |
| LPA: 5-classes; | 222 | 222 | -7190 | 14823 | 15579 | 14875 |
| LPA: 6-classes; | 222 | 264 | -7120 | 14768 | 15666 | 14830 |

| **Entropy** | **VLMR_PValue** | **LMR_PValue** | **BLRT_PValue** |
| --- | --- | --- | --- |
| NA | NA | NA | NA |
| 0.996 | 0.6095 | 0.6103 | 0 |
| 0.966 | 0.3056 | 0.3056 | 0 |
| 0.974 | 0.7704 | 0.7706 | 0 |
| 0.956 | 0.4054 | 0.4055 | 0 |
| 0.974 | 0.7617 | 0.7617 | 0 |

**Model probabilities**

**AIC**

| **Modnames** | **K** | **AIC** | **Delta_AIC** | **ModelLik** | **AICWt** | **LL** | **Cum.Wt** |
| --- | --- | --- | --- | --- | --- | --- | --- |
| LPA: 6-classes; | 264 | 14768 | 0 | 1 | 1 | -7120 | 1 |
| LPA: 5-classes; | 222 | 14823 | 55.25 | 1.007e-12 | 1.007e-12 | -7190 | 1 |
| LPA: 4-classes; | 180 | 14888 | 120.1 | 8.28e-27 | 8.28e-27 | -7264 | 1 |
| LPA: 3-classes; | 138 | 15122 | 353.8 | 1.467e-77 | 1.467e-77 | -7423 | 1 |
| LPA: 2-classes; | 96 | 15513 | 745.4 | 1.396e-162 | 1.396e-162 | -7661 | 1 |
| LPA: 1-classes; | 54 | 18380 | 3612 | 0 | 0 | -9136 | 1 |

**BIC**

| **Modnames** | **K** | **BIC** | **Delta_BIC** | **ModelLik** | **BICWt** | **LL** | **Cum.Wt** |
| --- | --- | --- | --- | --- | --- | --- | --- |
| LPA: 4-classes; | 180 | 15501 | 0 | 1 | 1 | -7264 | 1 |
| LPA: 5-classes; | 222 | 15579 | 78.05 | 1.127e-17 | 1.127e-17 | -7190 | 1 |
| LPA: 3-classes; | 138 | 15591 | 90.81 | 1.912e-20 | 1.912e-20 | -7423 | 1 |
| LPA: 6-classes; | 264 | 15666 | 165.7 | 1.037e-36 | 1.037e-36 | -7120 | 1 |
| LPA: 2-classes; | 96 | 15840 | 339.4 | 1.964e-74 | 1.964e-74 | -7661 | 1 |
| LPA: 1-classes; | 54 | 18564 | 3063 | 0 | 0 | -9136 | 1 |

**Technical summary of final model**

.

| **Mplus.version** | 8.4 |
| --- | --- |
| **Title** | LPA: 4-classes; |
| **AnalysisType** | mixture |
| **DataType** | INDIVIDUAL |
| **Estimator** | MLR |
| **Observations** | 222 |
| **NGroups** | 1 |
| **NDependentVars** | 20 |
| **NIndependentVars** | 0 |
| **NContinuousLatentVars** | 0 |
| **NCategoricalLatentVars** | 1 |
| **Parameters** | 180 |
| **ChiSqCategoricalPearson_Value** | 3059.21 |
| **ChiSqCategoricalPearson_DF** | 77968 |
| **ChiSqCategoricalPearson_PValue** | 1 |
| **ChiSqCategoricalLRT_Value** | 664.831 |
| **ChiSqCategoricalLRT_DF** | 77968 |
| **ChiSqCategoricalLRT_PValue** | 1 |
| **ChiSqMCARUnrestrictedPearson_Value** | 12.797 |
| **ChiSqMCARUnrestrictedPearson_DF** | 65620 |
| **ChiSqMCARUnrestrictedPearson_PValue** | 1 |
| **ChiSqMCARUnrestrictedLRT_Value** | 10.772 |
| **ChiSqMCARUnrestrictedLRT_DF** | 65620 |
| **ChiSqMCARUnrestrictedLRT_PValue** | 1 |
| **LL** | -7264.041 |
| **LLCorrectionFactor** | 1.2995 |
| **AIC** | 14888.08 |
| **BIC** | 15500.57 |
| **aBIC** | 14930.13 |
| **Entropy** | 0.974 |
| **T11_KM1Starts** | 100 |
| **T11_KM1Final** | 20 |
| **T11_KM1LL** | -7422.901 |
| **T11_VLMR_2xLLDiff** | 317.719 |
| **T11_VLMR_ParamDiff** | 43 |
| **T11_VLMR_Mean** | 1724.68 |
| **T11_VLMR_SD** | 1900.903 |
| **T11_VLMR_PValue** | 0.7704 |
| **T11_LMR_Value** | 316.357 |
| **T11_LMR_PValue** | 0.7706 |
| **BLRT_RequestedDraws** | Varies |
| **BLRT_KM1AnalysisStarts** | 100 |
| **BLRT_KM1AnalysisFinal** | 20 |
| **BLRT_KM1GenStarts** | 0 |
| **BLRT_KM1GenFinal** | 0 |
| **BLRT_KGenStarts** | 40 |
| **BLRT_KGenFinal** | 8 |
| **BLRT_KM1LL** | -7422.901 |
| **BLRT_2xLLDiff** | 317.719 |
| **BLRT_ParamDiff** | 43 |
| **BLRT_PValue** | 0 |
| **BLRT_SuccessfulDraws** | 5 |
| **AICC** | 16477.35 |
| **Filename** | 4-class.out |

**Subtypes based on combined data from midas and spsr (no controls)**

**Table of model fit**

| **Title** | **Observations** | **Parameters** | **LL** | **AIC** | **BIC** | **aBIC** |
| --- | --- | --- | --- | --- | --- | --- |
| LPA: 1-classes; | 221 | 79 | -5801 | 11761 | 12029 | 11779 |
| LPA: 2-classes; | 221 | 159 | -4495 | 9309 | 9849 | 9345 |
| LPA: 3-classes; | 221 | 239 | -4234 | 8947 | 9759 | 9002 |
| LPA: 4-classes; | 221 | 319 | -4076 | 8790 | 9874 | 8863 |
| LPA: 5-classes; | 221 | 399 | -3994 | 8785 | 10141 | 8877 |
| LPA: 6-classes; | 221 | 479 | -3905 | 8767 | 10395 | 8877 |

| **Entropy** | **VLMR_PValue** | **LMR_PValue** | **BLRT_PValue** |
| --- | --- | --- | --- |
| NA | NA | NA | NA |
| 1 | 0.0059 | 0.006 | 0 |
| 0.966 | 0.7794 | 100 | 2909 |
| 0.985 | 0.7668 | 100 | 6728 |
| 0.982 | 0.7901 | 0.7901 | 0 |
| 0.984 | 0.7629 | 100 | 6992 |

**Model probabilities**

**AIC**

| **Modnames** | **K** | **AIC** | **Delta_AIC** | **ModelLik** | **AICWt** | **LL** | **Cum.Wt** |
| --- | --- | --- | --- | --- | --- | --- | --- |
| LPA: 6-classes; | 479 | 8767 | 0 | 1 | 0.9999 | -3905 | 0.9999 |
| LPA: 5-classes; | 399 | 8785 | 18 | 0.0001235 | 0.0001235 | -3994 | 1 |
| LPA: 4-classes; | 319 | 8790 | 22.66 | 1.202e-05 | 1.202e-05 | -4076 | 1 |
| LPA: 3-classes; | 239 | 8947 | 179.7 | 9.482e-40 | 9.481e-40 | -4234 | 1 |
| LPA: 2-classes; | 159 | 9309 | 541.3 | 2.818e-118 | 2.818e-118 | -4495 | 1 |
| LPA: 1-classes; | 79 | 11761 | 2993 | 0 | 0 | -5801 | 1 |

**BIC**

| **Modnames** | **K** | **BIC** | **Delta_BIC** | **ModelLik** | **BICWt** | **LL** | **Cum.Wt** |
| --- | --- | --- | --- | --- | --- | --- | --- |
| LPA: 3-classes; | 239 | 9759 | 0 | 1 | 1 | -4234 | 1 |
| LPA: 2-classes; | 159 | 9849 | 89.78 | 3.2e-20 | 3.2e-20 | -4495 | 1 |
| LPA: 4-classes; | 319 | 9874 | 114.8 | 1.177e-25 | 1.177e-25 | -4076 | 1 |
| LPA: 5-classes; | 399 | 10141 | 382 | 1.124e-83 | 1.124e-83 | -3994 | 1 |
| LPA: 6-classes; | 479 | 10395 | 635.9 | 8.447e-139 | 8.447e-139 | -3905 | 1 |
| LPA: 1-classes; | 79 | 12029 | 2270 | 0 | 0 | -5801 | 1 |

**Technical summary of final model**

| **Mplus.version** | 8.4 |
| --- | --- |
| **Title** | LPA: 3-classes; |
| **AnalysisType** | mixture |
| **DataType** | INDIVIDUAL |
| **Estimator** | MLR |
| **Observations** | 221 |
| **NGroups** | 1 |
| **NDependentVars** | 20 |
| **NIndependentVars** | 0 |
| **NContinuousLatentVars** | 0 |
| **NCategoricalLatentVars** | 1 |
| **Parameters** | 239 |
| **LL** | -4234.474 |
| **LLCorrectionFactor** | 1.043 |
| **AIC** | 8946.947 |
| **BIC** | 9759.108 |
| **aBIC** | 9001.706 |
| **Entropy** | 0.966 |
| **T11_KM1Starts** | 100 |
| **T11_KM1Final** | 20 |
| **T11_KM1LL** | -4495.289 |
| **T11_VLMR_2xLLDiff** | 521.631 |
| **T11_VLMR_ParamDiff** | 80 |
| **T11_VLMR_PValue** | 0.7794 |
| **T11_LMR_Value** | 520.426 |
| **T11_LMR_PValue** | 0.7794 |
| **BLRT_RequestedDraws** | Varies |
| **BLRT_KM1AnalysisStarts** | 100 |
| **BLRT_KM1AnalysisFinal** | 20 |
| **BLRT_KM1GenStarts** | 0 |
| **BLRT_KM1GenFinal** | 0 |
| **BLRT_KGenStarts** | 40 |
| **BLRT_KGenFinal** | 8 |
| **BLRT_KM1LL** | -4495.289 |
| **BLRT_2xLLDiff** | 521.631 |
| **BLRT_ParamDiff** | 80 |
| **BLRT_PValue** | 0 |
| **BLRT_SuccessfulDraws** | 5 |
| **AICC** | 2909.052 |
| **Filename** | 3-class.out |

Supplement 3. **Number of subjects classified into each latent class**

|  |  | **TTM** | **SPD** | **TTM+SPD** | **Controls** |
| --- | --- | --- | --- | --- | --- |
|  | |  |  |  |  |
| **Each latent class as a function of clinical subtype using the MGH-HPS + MIST-A-R.** | | | | | |
|  | |  |  |  |  |
| **Latent class 1 (TTM-absent/controls)** | | 0 | 62 | 2 | 57 |
| **Latent class 2 (TTM subtype 1)** | | 14 | 8 | 5 | 0 |
| **Latent class 3 (TTM subtype 2)** | | 58 | 4 | 19 | 0 |
| **Latent class 4 (TTM subtype 3)** | | 35 | 1 | 14 | 0 |
|  | |  |  |  |  |
| **Each latent class as a function of clinical subtype using the SPS-R + MIDAS.** | | | | | |
| **Latent class 1 (SPD-absent/controls)** | | 64 | 0 | 0 | 51 |
| **Latent class 2 (SPD subtype 1)** | | 8 | 73 | 32 | 2 |
| **Latent class 3 (SPD subtype 2)** | | 28 | 8 | 8 | 5 |
|  | |  |  |  |  |

TTM: Trichotillomania cases, SPD: Skin Picking Disorder cases, TTM+SPD: Trichotillomania+Skin Picking Disorder cases.
